# Supplementary material for: Identification of Reassortment of Orthotospovirus citrullomaculosi in Jiangxi Province, China
Source: Viruses. 2025 Oct 31;17(11):1448. doi: 10.3390/v17111448 (PMC12656826; doi:10.3390/v17111448)
Supplement: Supplementary file 1 [file viruses-17-01448-s001.zip › viruses-3903258-supplementary.pdf]

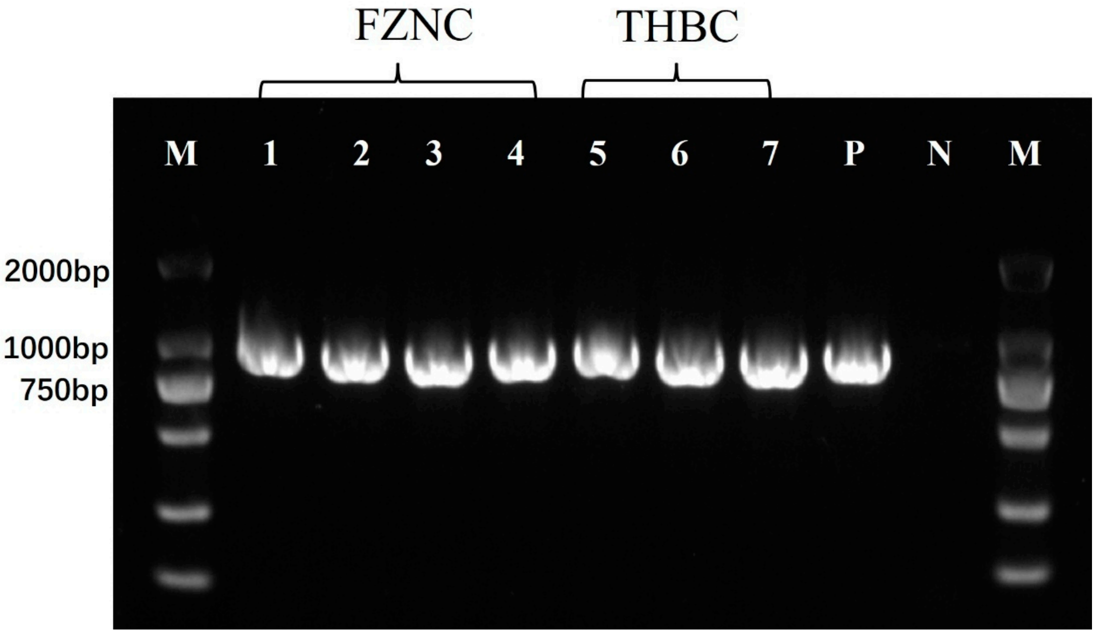

Figure S1. Agarose gel electrophoresis of RT-PCR products amplified with WSMoV-specific primers M: DL2000 marker, lane 1-4：FZNC1-4 samples from Nancheng county, Fuzhou city; lane 5-7: THBC1-3 samples from Taihe county, Ji'an city, P: positive control, N: negative control.

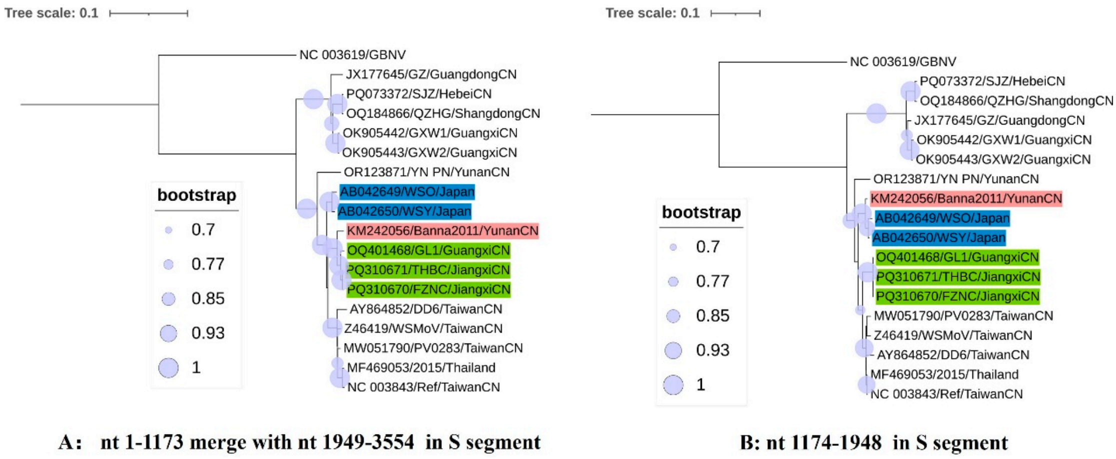

Figure S2. Phylogenetic analysis of non-recombinant regions in S segment. (A) Phylogenetic tree based on nt 1–1173 merge with nt 1949-3554 sequences; (B) Phylogenetic tree based on nt 1174–1948 sequences. Accession number, isolate name, and collected location are provided in nodes. Isolates with red, green, and blue backgrounds represent recombinant isolates, minor parents and major parents in the interspecific recombinant event, respectively.

Table S1 The details of primer pairs in this study

Primer	Reference genome accessions	Location	Sequences (5′-3′)	Production size
WS-F	NC_003843	2640-2670	GTTAYACTTC CAARGAAGTGTCTRGGYTTT G	∽890 bp
WS-R		3533-3503	AGAGCAATCGAGGCGCTAATAWAATCAGTTC	
CGCPF	NC_001801	5683-5707	CGTGG TAAGC GGCATTCTAAACCTC	∽650 bp
CGCPR		6338-6317	CCGCAAACCAATGAGCAAACCG	
WS-L4826F	PQ310674	4824-4842	TTTGAGGAAAGGTTGCCC	∽1050bp
WS-L5869R		5850-5869	TTAGGGATAGAAGCATTGGC	
WS-M2347F	PQ310672	2347-2370	AAAGTGGATGGAACTGATAACGGG	∽930bp
WS-M3278R		3257-3278	CGAAATGGCTGTAACTCCCTTG	
